# The Book World of Medicine and Science

**Published:** 1893-11-25

**Authors:** 


### The Book World of Medicine and Science.

The Disease of Inebriety from Alcohol, Opium, and other Narcotic Drugs; its Etiology, Pathology, Treatment, and Medico-Legal Relations. Arranged and compiled by the American Association for the Study and Cure of Inebriety. (Bristol: John Wright and Co., Stone Bridge. London: Simpkin, Marshall, Hamilton, Kent, and Co., Limited. 1893. Price six shillings.)

Many books, great and small, are constantly issuing from the Press on the "drink question." Some of these are wise and some are foolish, but almost all are incomplete and one-sided. This volume, issued by the American Association for the Study of Inebriety, is certainly one of the fullest and most interesting that we have ever met with. The inquiries of the authors—for we believe that in spite of its unity and sequence this volume is the work of several hands—have not been confined, as most are, to alcoholism. Due consideration has been given also to those other intoxicants in which the more sensitive and refined portions of the community are tempted to indulge—opium, ether, cocaine, chloroform, and even coffee and tea. That these two last beverages, popularly regarded as teetotal, are included among intoxicants, shows the thorough-going nature of the investigation, though, perhaps, it may cause the book to be estimated at less than its just value by those tea drinkers who "compound for sins they are inclined to" by—very maledictory treatment of all others.

The American origin of the book is most clearly shown in the attribution of inebriety chiefly to the hurry of a restless age. This is natural in the citizens of a country where, as Dr. Talmage says, "We were born in a hurry, live in a hurry, die in a hurry, and are driven to Greenwood Cemetery on a trot." While this may be true, however, we think our authors go too far when they protest against the carrying of watches as conducive to neurosis, even though "a distinguished alienist" declares that "a look at one's watch, when an appointment is near, sensibly accelerates the heart's action, and is correlated to a definite loss of nervous energy." It is to be feared that we cannot afford to be habitually unpunctual, even to save our nerves. We English, however, are in less dangerous case than our transatlantic brethren, and one doctor pays us the left-handed compliment of saying that it is worth an ocean voyage to see how Englishmen can drink.

With regard to treatment, the one thing recommended is restraint for a sufficiently long period, with educational and industrial training as well as hygienic treatment. "Not far away in the future," says this volume, "inebriety will be regarded as small-pox cases are now in every community. The inebriate will be forced to go into quarantine, and be treated for his malady until he recovers." The shortest period of quarantine is to be one year, but some cases should be secluded for three, five, or ten years, and incurables might even be condemned—one can use no other word—for life. It cannot be doubted that such a system would have a marked effect on national inebriety, not merely in reforming individual drunkards, but in preventing, or at least limiting, by this seclusion, the transmission of hereditary tendencies.

With regard to inebriety from narcotics, it is pointed out that mere withdrawal of the drug cannot be regarded as cure. The disease or pain which first induced the indulgence must be dealt with, nutrition and digestion must be treated, the general health and surroundings, physical and moral, duly considered. In opium inebriety confinement to bed during the early part of the treatment is recommended, as tending to promote nutrition and lessen the tax on the nervous system, while heart tonics may be given as substitutes for the stimulation of the opium. Tea seems to be, when taken in excess, an intoxicant of a peculiarly fatal character. So much may be inferred from the case of a boy who suffered from delirium induced by excessive tea drinking, and chewing the dry leaf. Cured of this, he took to coffee in excess, and when this was prevented, fell back on opium. It will be seen from these extracts that the book is a peculiarly interesting one, and while one may not agree with all the conclusions of

**THE BOOK WORLD OF MEDICINE AND SCIENCE**—*continued.*

the authors, it must be admitted that they have collected a large amount of invaluable information on the subject they deal with in this book.

Sanitary Science and Preventive Medicine. (Transactions of the Sanitary Institute, Vol. XIII., 1892. Edward Stanford.)

In the section of Sanitary Science and Preventive Medicine an address on the Causes and Prevention of Cholera was delivered by the President, Professor Lane Notter, in which he discussed recent investigations on the spread of the disease and precautions founded on them. A paper of a somewhat alarmist nature was read by Mr. Oldfield, who contended that tuberculosis was largely spread by the consumption of the flesh of tuberculous animals, and that vegetarianism offered the only solution of the difficulty. He stated that tuberculosis in animals was extremely common, and that meat inspection was inefficient as regards this disease. In the discussion which followed, there was a general feeling that the writer had not maintained his thesis. Dr. Armand Ruffer, while admitting the danger to children from milk, stated that he had not come across five adults in which he could trace death to tubercular food. He stated also that phthisis was chiefly propagated by direct contact with phthisical patients. Sir Charles Cameron also directed attention to the fact that tubercle was frequent in many districts where the people were vegetarians or used only pickled bacon as meat. A resolution in favour of public abattoirs instead of private slaughter-houses was carried unanimously.

An interesting contribution to our knowledge as to the duration of the immunity conferred by vaccination was made by Veterinary-Captain Smith in a paper based on the returns of the Army Vaccine Institute. He showed that 68 per cent. of the re-vaccinations in persons entering the army were successful, and therefore that that proportion of young men between the ages of 18 and 20 years were probably susceptible of small-pox, while others were so in a less degree, as shown by less perfect vesicles, and only a small percentage could not be vaccinated. Re-vaccination was strongly advocated at the age of twelve years.

The advantages of "earth-to-earth" burial were duly set forth in a popular manner by the Rev. F. Lawrence, as also the evils of the present system of burial. As might be expected, many of those present expressed themselves in favour of cremation, though it was explained that the system supported by the writer of the paper was put forward in deference to the feelings of those who were opposed to cremation, in order to meet the sanitary defects of the existing methods of burial. Anyone having even an elementary knowledge of the chemical changes which take place in the dead body will admit that burial in strong and durable coffins is irrational, both as regards the dead and the living. But for the sentiment induced by long-prevailing custom it is probable that the cremation movement would have extended far more rapidly than has been the case, and to those who thus feel the influence of tradition the perishable coffin offers a means of burial with reverence to the dead and safety to the living.

A paper was read by Sir Charles Cameron on Infantile Mortality, in which the causes of the high death-rate which prevails among the children of our manufacturing towns. As showing the influence of social condition, the writer stated that in Dublin the death-rate in 1891 for children under five years of age in the professional and independent classes was 6 per cent. of the total deaths in the class, while among the porters, labourers, &c., the proportion was 42 per cent. In the discussion it was pointed out that in rural districts near London and other cities it was often difficult for the inhabitants to obtain milk for their children, and that artificial foods were more largely used in the country than in town.

Papers were also read on the route of cholera in 1892, and the precautions to be taken at harbours with regard to cholera and other infectious diseases; also on the physical conditionof children, with copious statistics.

